# Molecular basis of classic galactosemia from the structure of human galactose 1-phosphate uridylyltransferase

**DOI:** 10.1093/hmg/ddw091

**Published:** 2016-03-22

**Authors:** Thomas J. McCorvie, Jolanta Kopec, Angel L. Pey, Fiona Fitzpatrick, Dipali Patel, Rod Chalk, Leela Shrestha, Wyatt W. Yue

**Affiliations:** ^1^Structural Genomics Consortium, Nuffield Department of Clinical Medicine, University of Oxford, Oxford OX3 7DQ , UK; ^2^Department of Physical Chemistry, Faculty of Sciences, University of Granada, Granada E-18071, Spain

## Abstract

Classic galactosemia is a potentially lethal disease caused by the dysfunction of galactose 1-phosphate uridylyltransferase (GALT). Over 300 disease-associated *GALT* mutations have been reported, with the majority being missense changes, although a better understanding of their underlying molecular effects has been hindered by the lack of structural information for the human enzyme. Here, we present the 1.9 Å resolution crystal structure of human GALT (hGALT) ternary complex, revealing a homodimer arrangement that contains a covalent uridylylated intermediate and glucose-1-phosphate in the active site, as well as a structural zinc-binding site, per monomer. hGALT reveals significant structural differences from bacterial GALT homologues in metal ligation and dimer interactions, and therefore is a zbetter model for understanding the molecular consequences of disease mutations. Both uridylylation and zinc binding influence the stability and aggregation tendency of hGALT. This has implications for disease-associated variants where p.Gln188Arg, the most commonly detected, increases the rate of aggregation in the absence of zinc likely due to its reduced ability to form the uridylylated intermediate. As such our structure serves as a template in the future design of pharmacological chaperone therapies and opens new concepts about the roles of metal binding and activity in protein misfolding by disease-associated mutants.

## Introduction

Galactose (Gal) is an essential monosaccharide within the human body, with the Leloir pathway being its principal metabolic route (1,2). The Leloir pathway consists of four enzymes, namely galactose mutarotase (GALM), galactokinase 1, (GALK1), galactose 1-phosphate uridylyltransferase (GALT) and UDP-galactose 4′-epimerase (GALE) (Supplementary Material, Fig. S1A). Together these enzymes convert galactose, from dietary and endogenous sources, into glucose for glycolysis, but also inter-convert uridine diphosphate (UDP) hexoses that are used in the formation of glycogen (3) and glycoconjugates (4). Additionally, GALT and GALE are involved in the metabolism of UDP-*N*-acetyl-hexose-amines (5,6), which are substrates of glycosyltransferases (7,8), and are highly important structural elements of glycosaminoglycans (9). The essentiality of this pathway is underlined by the group of genetic disorders known as galactosemia due to defects on the GALK1, GALT or GALE enzymes (10).

GALT (EC 2.7.7.12) reversibly converts galactose 1-phosphate (Gal-1-P) and UDP glucose (UDP-Glc) into glucose 1-phosphate (Glc-1-P) and UDP galactose (UDP-Gal) (Supplementary Material, Fig. S1A). Inherited mutations on the *GALT* gene results in autosomal recessive, classic galactosemia (or type I galactosemia; OMIM #230400) (11). Newborn sufferers of classic galactosemia typically present jaundice, cataracts, hepatomegaly and in severe cases, death (12). The only available treatment is a strict dietary restriction of galactose, which treats most symptoms in early life. Even with dietary adherence older sufferers however tend to show neurocognitive disabilities in learning and speech, with ovarian failure commonly found in female galactosemics (13). Hallmarks of classic galactosemia are elevated levels of the metabolite galactose 1-phopshate (14), reduced levels of UDP-hexoses (15) and disturbed glycosylation (16–19) all of which are thought to contribute to disease aetiology. To date 336 mutations have been reported in the GALT database (http://www.arup.utah.edu/database/galt/GALT_display.php, last accessed January 2016), of which ∼60% represent missense changes (20).

No crystal structure of human GALT (hGALT) has been reported to date, however multiple *Escherichia coli* structures (eGALT, bearing 55% sequence identity to human protein) and accompanying studies have given insight into the enzymatic function (21). GALT is a dimeric β-strand rich protein, belonging to the histidine triad superfamily (22). The eGALT structure confirms biochemical studies showing a two-step ‘ping-pong’ mechanism that involves an enzyme-bound uridylylated intermediate (23–25). In the first step, hydrolysis of the UDP-sugar substrate results in a phospho-histidine bond between uridine monophosphate (UMP) and His166 (His186 in hGALT) from the active site motif ‘HPH’ (residues 164–166), with the release of hexose 1-phosphate (Supplementary Material, Fig. S1B). In the second step, this covalent uridylylated intermediate is attacked by an incoming hexose-1-phosphate, forming the UDP-sugar product. GALT is a metallo-protein, with the eGALT structure showing zinc and iron binding at two different sites. The zinc-binding site (Cys52, Cys55, His115, His164) has been shown to be important for catalysis by structurally stabilising the active site. The iron-binding site on the other hand (Glu182, His281, His299, His301) has been suggested to stabilise the structure of the protein (26). Interestingly the zinc-binding site residues are not all conserved in hGALT (Asn72, Cys75, Ser135, His184), but it has been reported that hGALT binds zinc (27). The eGALT iron-binding site has the ability to bind other divalent metal ions, suggesting the same site in hGALT binds zinc (26). In the absence of experimental structures, a homology model for hGALT was published (28,29) to gain insight into the structural effects of disease-associated mutations. Unfortunately, the model does not take into account the metal stoichiometry of hGALT. In addition, there are instances where there is no clear correlation between the predicted structural effect of disease-associated mutants and their functional effect on the protein (29–31). These observations demonstrate that the homology models are not fully accurate and that a crystal structure of hGALT is much needed, which is the subject of this study.

## Results

### Recombinant hGALT exists in equilibrium between apo and uridylylated states

Recombinant hGALT (harbouring the p.Asn319Asp polymorphism, see Materials and Methods) was expressed in *E. coli* and the purified protein resolved in intact mass spectrometry as two species at 43 450 and 43 755 Da (Supplementary Material, Fig. S1B), with the mass difference (305 Da) attributed to a UMP moiety. Reasoning that the two species represent the apo (apo-hGALT) and uridylylated (UMP-hGALT) enzymes in equilibrium, as reported *in vivo* (32), we incubated as-purified hGALT with Glc-1-P or UDP-Glc, in hopes of obtaining apo-hGALT or UMP-hGALT, respectively. An increase of peak signal for the expected product species was observed, indicating that the recombinant enzyme is active. The active site variant p.His186Ala appeared only as the apo species with no indication of uridylylation even upon incubation with UDP-Glc (Supplementary Material, Fig. S1C), confirming that His186 is the site of uridylylation.

### hGALT structure and dimer interface

**Figure 1.**
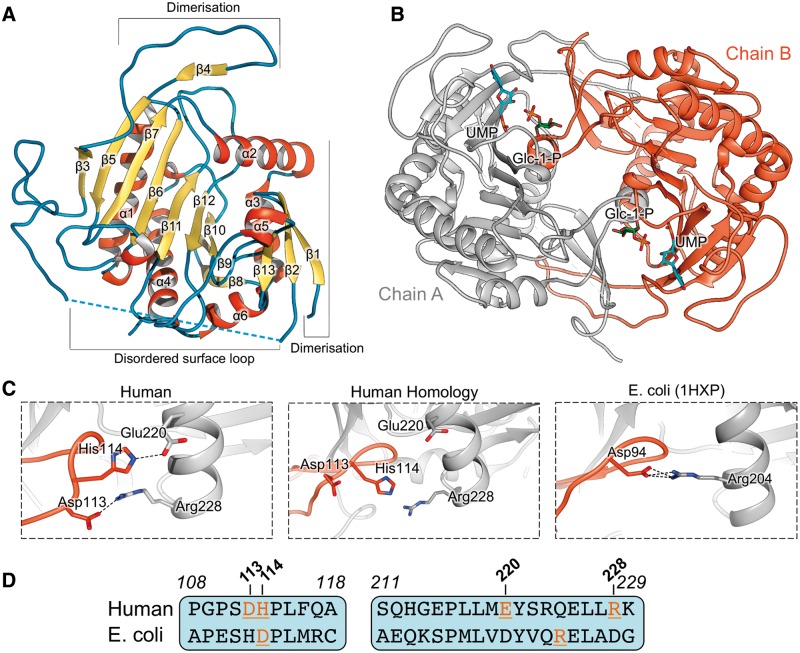
Crystal structure of uridylylated hGALT at 1.9 Å resolution. (**A**) Cartoon representation of hGALT structure (chain A) showing secondary structure elements. Regions involved in dimerisation are highlighted along with the disordered region that presumably interacts with UDP-hexoses. (**B**) View of the hGALT dimer along the 2-fold symmetry axis. UMP and Glc-1-P are represented as sticks. (**C**) Analysis of the inter-chain salt bridge interactions of the eGALT and hGALT structures, as well as the hGALT homology model, showing important differences. Our hGALT structure reveals two salt bridges, Asp113^B ^− Arg228^A^ and His114^B ^− Glu220^A^, whereas only one salt bridge is found in eGALT between Asp94^B^ and Arg204^A^. No interaction between any of these residues was observed in the homology model. (**D**) Sequence alignment suggests the differences in dimeric interaction patterns between eGALT and hGALT are due to a lack of sequence conservation of these residues, resulting in the inaccurate prediction of the homology model.

hGALT was crystallized in the presence of excess UDP-Glc, and the structure was determined to 1.9 Å resolution (Protein Data Bank code 5IN3) by molecular replacement using the eGALT structure (55% sequence identity) (Supplementary Material, Table S1). The hGALT protomer (Fig. 1A) adopts the same structural fold as eGALT (C^α^-RMSD 0.7 Å, Supplementary Material, Fig. S3A), and consists of a central nine-stranded β-sheet flanked on both sides by five α-helices and a small three-stranded β-sheet. Residues 49–63, corresponding to a surface-exposed loop involved in UDP-hexose binding, are disordered in the structure, suggesting its mobility in the absence of this ligand. hGALT is an obligate dimer, where each active site is formed by both dimeric subunits (Fig. 1B). This interface buries ≈ 3100 Å^2^ of protein surface and is contributed by 17 hydrogen bonds and two salt bridges. The two salt bridges (Asp113^B ^− Arg228^A^ and His114^B ^− Glu220^A^) are present at the end of a dimerisation loop (residues 106–122) and stabilise this structure against helix α3 (Fig. 1C, left). This results in an inter-chain β-sheet formed by strands β1 and β2 from one chain, and β4 from the other (Fig. 1B).

Our hGALT structure reveals significant discrepancies at the side chain level with the eGALT structure due to sequence changes, and thus with the previously reported hGALT homology model that was built using eGALT as template. These discrepancies are particularly pronounced at the dimer interface, where inter-chain hydrogen bonds and salt bridges show the least agreement among the structures (Supplementary Material, Fig. S4). For example, while hGALT has two inter-dimer salt bridges as described above, only one salt bridge is present in the eGALT dimer interface (Fig. 1C, middle) due to amino acid changes at the equivalent positions (Fig. 1D). The implication is an incorrect prediction in the hGALT homology model, where no interaction occurs between these residues (Fig. 1C, right). In another example, an inter-dimer salt bridge was predicted in the hGALT homology model between Glu40^B^ and Arg201^A^, which was not present in our crystal structure due to the orientation of the side-chains of these residues (Supplementary Material, Fig. S3B). Our experimentally determined hGALT structure therefore provides a better framework to understand the structural effects of the majority of disease-associated variants (see section ‘Structural basis of classic galactosemia variants’).

### Uridylylation induces conformational changes to hGALT

**Figure 2.**
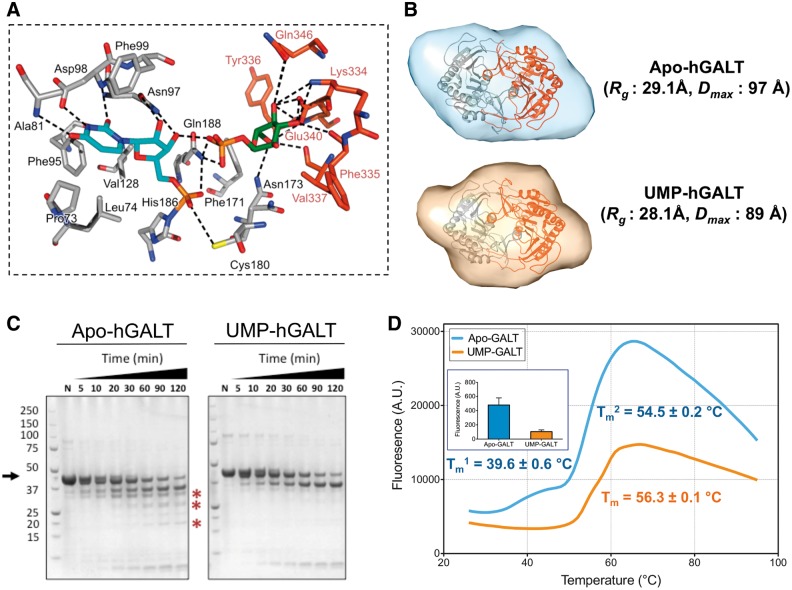
Uridylylation induces a global contraction of hGALT. (**A**) Active site residues involved in UMP and Glc-1-P binding. (**B**) *Ab initio* apo-hGALT and UMP-hGALT envelopes as calculated by GASBOR. (**C**) Native proteolysis of apo-hGALT and UMP-hGALT showing the compaction is subtle. Apo-hGALT shows lower MW degradation products at later times around 30 and 20 kDa in size (asterisks). (**D**) Representative thermal shift curves show differences between apo-hGALT and UMP-GALT. (*T*_m_ calculated from at least three replicates.) Inset is the surface hydrophobicity of both species as determined by Sypro orange binding at room temperature.

The hGALT structure represents the uridylylated enzyme with a covalent linkage between UMP and His186 at the active site. The UMP binding site (Fig. 2A) is largely similar to that of eGALT (24), with the exception of two residues Pro73 and Ala81 (Phe53 and Val61 in *E. coli*, respectively) which form non-polar contacts with the uridine moiety. Electron density also revealed Glc-1-P in both active sites of the dimer, modelled at 70% occupancy. Thus our structure represents a ternary complex in the reaction path of hGALT, after hydrolysis of the UDP-Glc substrate. The Glc-1-P binding site (Fig. 2A) is formed by Lys334, Phe335, Val337, Tyr339, Glu340 and Gln346 from one chain, along with Gln188 and Asn173 from the other chain. The Glc-1-P binding mode is similar to that of the eGALT structure bound with UDP-Glc (24) with notable differences being the lack of interactions with Arg48, Arg51, Gly179 and Ser181 (Arg28, Arg31 Gly159 and Ser161 in *E. coli*, respectively) that are likely due to the position of the UMP phosphate. Compared to the bacterial structure, this phosphate is displaced by 2.5 Å in hGALT due to a covalent bond with His186. It is of note that the side-chains of Asn173 and Glu340 demonstrate occupancy of two different orientations in both active sites. Comparison of both bacterial structures with UDP-Glc or UDP-Gal bound have shown that the equivalent residue of Glu340, Glu323, moves depending on the position of the 4′-hydroxyl group of the sugar (24). The observed dual occupancy suggests a similar role for this residue in hGALT.

Our structure, along with the bacterial structures, suggests that the loop harbouring residues 49–63 becomes ordered only when UDP-Glc/Gal is bound to the active site. However, little is known if uridylylation results in any changes in conformation. To address this, we performed small angle X-ray scattering (SAXS) on apo- and UMP-hGALT. Both samples exhibited properties of a globular protein (Fig. 2B), although uridylylation decreases the radius of gyration (*R_g_*) by 1 Å and maximal intraparticle dimension (*D*_max_) by 8 Å, consistent with a more compact structure for UMP-hGALT compared to apo-hGALT. *Ab initio* envelopes calculated for both samples (Fig. 2B) fit one dimer of hGALT, although the crystal structure fits better into the UMP-hGALT envelope. We pursued further biophysical experiments to demonstrate that uridylylation induces structural change to the protein and reduces its flexibility. First, native proteolysis revealed more degradation products with apo-hGALT than UMP-hGALT (Fig. 2C, asterisks). Second, thermal stability measurements using differential scanning fluorimetry (DSF) showed a two-phase unfolding curve for apo-hGALT (*T*_m_^1^ of 39.6 °C and *T*_m_^2^ of 54.5 °C). In contrast UMP-hGALT unfolds at a higher apparent *T*_m_ (56.3 °C). On closer inspection this unfolding curve appears to be biphasic, although the closeness of the two transitions prevents an accurate determination of their respective *T*_m_s (Fig. 2D). Lastly, measurements of the intrinsic hydrophobicity showed that UMP-hGALT is more compact than apo-hGALT (Fig. 2D, inset). We postulate that the structural change due to uridylylation could be due to decreasing the flexibility of the loop involved in uracil binding (aa 76–90) though changes in global flexibility are likely to occur as well.

### Zinc binding stabilises hGALT

**Figure 3.**
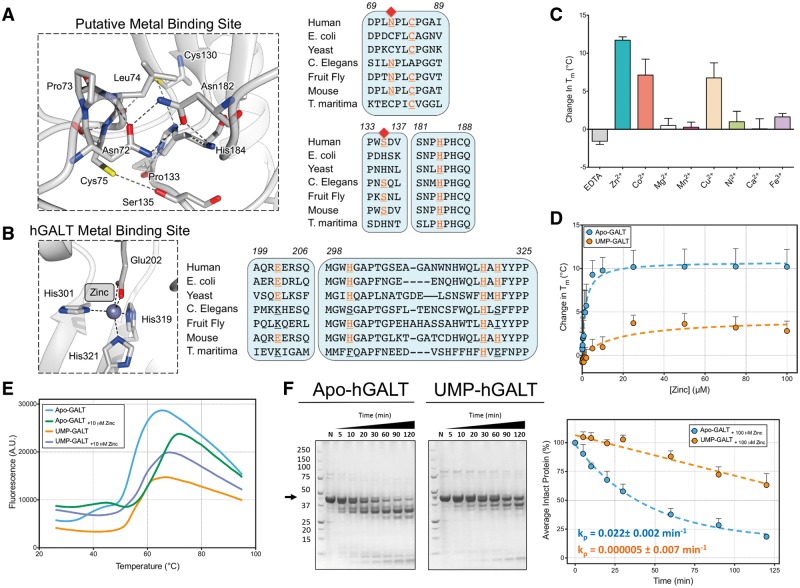
hGALT contains one divalent metal binding site with a preference for zinc. (**A**) Left, the structural environment of the putative metal site shows that unlike eGALT, hGALT does not bind zinc in this region due to the substitution of Asn72 and Ser135. Right, these residues are not strictly conserved across all GALT orthologues allowing us to predict that, along with eGALT, yeast and Thermotoga maritima (*T. maritima*) GALT could bind zinc in this region. (**B**) Left, the structural environment of the observed Zn^2+ ^binding site in hGALT shows residues that coordinate the metal ion. Right, these residues are not strictly conserved in all orthologues, which suggests metal binding at this site is not an invariant feature of GALT enzymes. (**C**) DSF analysis of apo-hGALT in the presence of various divalent metals confirms the plasticity of this site. The maximal *T*_m_^2^ change observed with Zn^2+ ^suggests this is the preferred ligand. (**D**) Thermal shift dose responses of apo- and UMP-hGALT show differing thermodynamic responses to Zn^2+^, with UMP-hGALT being thermally less stablised than apo-hGALT. (**E**) Representative DSF unfolding curves of apo- and UMP-hGALT in the presence of Zn^2+ ^showing only *T*_m_^2^ of apo-hGALT to be altered by Zn^2+ ^binding. (**F**) Native proteolysis of apo-hGALT and UMP-hGALT in the presence of Zn^2+ ^again highlights the differences between these two species. Here, UMP-hGALT is significantly more protected from degradation than apo-hGALT.

A key discovery from our structure is the presence of only one metal binding site per hGALT monomer, as opposed to two in eGALT. A putative site, which contains Zn^2 + ^and involves His164 of the ‘HPH’ motif in eGALT (23–25), shows no evidence of a divalent metal bound in hGALT. This is due to the replacement of residues Cys52 and His115 in eGALT to Asn72 and Ser135, respectively, in hGALT (Fig. 3A). Here, hydrogen bonds between Asn72 and Asn182, and between Cys75 and Ser135, replace the Zn^2 + ^interactions. Indeed the Zn^2+^-coordinating residues of eGALT are not conserved across GALT orthologues, suggesting that this site is not important for activity and stability (Fig. 3B, left). In contrast, the observed hGALT metal site is located ≈ 20 Å away from the active site, and contributed by residues Glu202, His301, His319 and His321 (Fig. 3B). A divalent Zn^2+ ^ion with 50% occupancy was modelled into the structure, based on fluorescence emission at the Zn^2+ ^absorption edge (E = 9.6586 keV, data not shown). Alignment of these Zn^2+^-coordinating residues (Fig. 3B, right) suggests that this metal site is conserved in some (e.g. mouse, yeast, *E. coli* GALT), but not all organisms. In the eGALT structures (23–25), Fe^2+ ^ion was modelled into this site. To confirm our absorption study we analysed the metal ion preference of hGALT using DSF (Fig. 3C; Supplementary Material, Fig. S7), in which Zn^2+ ^exhibited the strongest thermal stabilization of apo-hGALT (Δ*T*_m_^2 ^= 11.7 °C), followed by Co^2+ ^(Δ*T*_m_^2 ^= 7.1 °C) and Cu^2+ ^(Δ*T*_m_^2 ^= 6.7 °C). Dose-response curves suggested that Zn^2+ ^binding to hGALT is specific (Fig. 3D), but affects the apo enzyme (Max *T*_m_^2 ^= 66.9 °C) more so than uridylylated hGALT (Max *T*_m_ = 58.0 °C). *T*_m_^1^ of apo-hGALT did not appear to change in the presence of Zn^2+^, though its low signal made it difficult to interpret any effects in some cases (Fig. 3E). In addition, the low Zn^2+ ^occupancy in our structure suggested loss of this ion during the purification process, and prompted us to repeat native proteolysis of the two hGALT species in the presence of this metal. This considerably pronounced the structural differences between the two protein species, where UMP-hGALT is much more resistant against proteinase K than apo-hGALT (Fig. 3F). Altogether our findings suggest hGALT contains one Zn^2+ ^binding site, which confers stability to the protein.

### Structural basis of classic galactosemia variants

As of January 2016, 336 mutations are annotated in the *GALT* database, with 178 being missense (20). While the nonsense, indel and splice-site mutations will likely result in truncated or misfolded protein with altered function, the effects of missense mutations are more difficult to predict. Therefore, we structurally mapped (Supplementary Material, Fig. S8) and categorized (Supplementary Material, Table S1) the resulting variants into possible molecular defects of substrate binding, metal binding, dimerisation and folding. Overall, variants that potentially destabilise and misfold the hGALT protein are most prevalent. They include amino acid changes expected to alter protein flexibility (i.e. p.Pro36Leu, p.Gly83Val/Arg, p.Pro166Ala, p.Leu217Pro), cause steric clashes (i.e. p.Cys130Tyr, p.Val168Leu, p.Ala276Asn, p.Leu289Phe/Arg), create cavities (i.e. p.Val125Ala, p.Leu226Val, p.Trp154Gly, p.Met336Leu) and remove hydrogen bonds (i.e. p.Arg67Cys, p.Glu220Lys, p.Gln317Arg/His, p.Arg328His). The second most represented variant group alters dimer interactions such as inter-chain salt-bridges (p.Asp113Asn, p.His114Leu and p.Glu220Lys). Variants that alter substrate binding were the third most represented (i.e. p.Phe95Leu, p.Asp98Asn/His, p.Cys180Phe, p.His186Tyr, p.Lys334Arg), followed by variants that alter metal binding (p.Glu202Lys, p.His319Gln and p.His321Tyr). In addition, a few variants located on the protein surface showed no apparent effect on the protein structure, and are referred to as polymorphisms (p.Asp28Asn/His/Tyr, p.Asp113Asn, p.Tyr165His, p.Glu291Lys/Val, p.Ala330Val and p.Asp314Asn).

**Figure 4.**
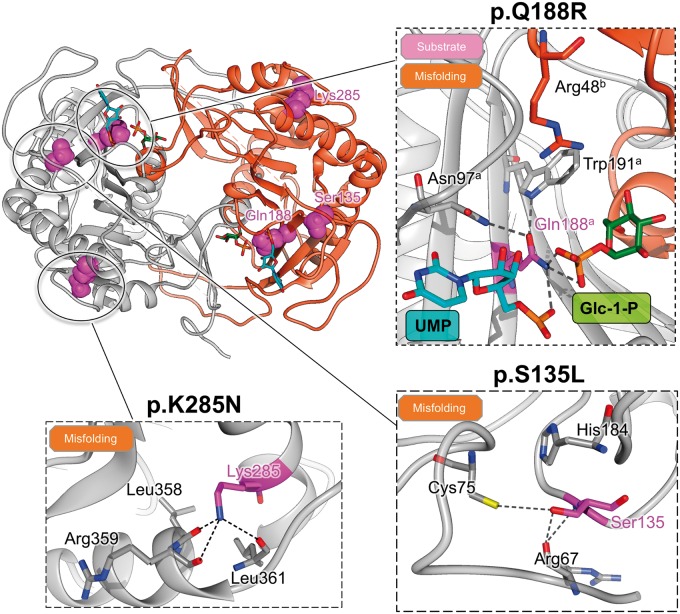
Structural basis of the most common hGALT variants. Structural mapping of the three most common variants gives a molecular explanation for their effects p.Ser135Leu is a misfolding variant as it removes hydrogen bond interactions and introduces a larger side-chain proximal to the active site; p.Lys285Asn is also a misfolding variant as it removes a number of hydrogen bonds and likely destabilises the enzyme; p.Gln188Arg is present at the active site and likely alters interactions with the substrate and the surrounding residues. Its proximity to the positively charged residue Arg57^B^ in the opposite chain of the dimer also suggests the potential for a misfolding affect.

Our structure also allows a detailed molecular view of the three variants accounting for the majority of classic galactosemia cases, namely p.Ser135Leu, p.Gln188Arg and p.Lys285Asn (Fig. 4). The p.Lys285Asn variant, commonly detected in Europeans (33), removes three hydrogen bonds involving helix α_6_, likely destabilising the protein. This agrees with our low recombinant yield for this variant (Supplementary Material, Fig. S2), although the purified protein is active (Supplementary Material, Fig. S1E). The p.Ser135Leu variant, exclusively found in African ethnicities (27,34), removes interactions with Cys72 and Arg67, and introduces a more hydrophobic and bulkier residue close to the site of uridylylation, His186. Previous modelling offered no structural explanation for this variant (29), and hGALT metal binding was shown to be not affected (27,34). The most common galactosemia variant (∼60% of patients), p.Gln188Arg, affects the active site residue Gln188 that interacts with the phosphate and ribose moieties of the covalently attached UMP, along with the phosphate moiety of the hexose-1-phosphate. Additionally, it forms interactions with Asn97 and Trp191 within the same chain of the dimer. These findings agree with previous homology models (28,29,31,35) which predicted an altered hydrogen-bonding pattern with the substrate explaining its low activity. However, there are contrasting hypotheses of under- (35) or over-stabilisation of the covalent intermediate (28,29,31). In addition, charge repulsion with the residue Arg48 from the opposite chain of the dimer has been suggested as a possible cause for this mutant’s partial dominant negativity (28).

### Molecular basis of the p.Gln188Arg dysfunction

**Figure 5.**
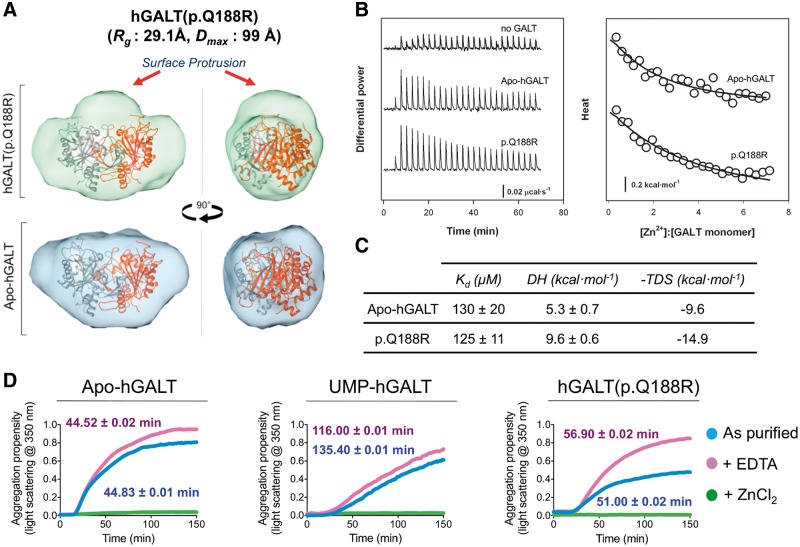
Biophysical characterisation of p.Gln188Arg. (**A**) Comparison of the hGALT(p.Q188R) and apo-hGALT envelopes as calculated by GASBOR. Both envelopes can fit one hGALT dimer structure, however hGALT(p.Q188R) shows additional density at the dimer interface suggesting a structural change in the surface of this mutant. (**B**) Titrations and (**C**) ITC parameters show that this structural change is not accompanied by a change in the variant’s ability to bind Zn^2+^. (**D**) Turbidity measurements showing the propensity to aggregate at 37 °C of apo-hGALT, UMP-hGALT and hGALT(p.Q188R). Values shown are t_1/2_, determined as the time to reach half maximal amount of aggregation.

To experimentally determine the molecular basis of the p.Gln188Arg defect, we characterized the recombinant variant protein. MS of as-purified hGALT(p.Q188R) revealed only the apo enzyme at 43 480 Da (Supplementary Material, Fig. S1D). Overnight incubation with UDP-Glc yielded partial uridylylation representing ≈8% of total protein, compared to 81% of hGALT (Supplementary Material, Fig. S1F). Therefore hGALT(p.Q188R) causes an under-stabilisation and/or lower catalytic activity, as demonstrated by an enzymatic study on this variant (35). We also observed that hGALT(p.Q188R) is more susceptible to proteolysis than both apo-hGALT and His186Ala variant, indicative of a structural defect (Supplementary Material, Fig. S9A). Consistent with this, SAXS analysis of hGALT(p.Q188R) showed a broader *P(r)* function and larger intra-particle dimension (*R_g_*_:_ 29.1 Å, *D*_max_: 99 Å) than apo-hGALT (Supplementary Material, Fig. S6). The *ab initio* envelope (Fig. 5A) shows a protrusion along one axis at the dimer interface, resulting in a less compact shape. The protrusion appears to be caused by an altered dimer interface, and we speculate that this could be due to a local rearrangement of the surface loop residues 48–63, which is involved in UDP-hexose binding and disordered in our structure. Furthermore, the p.Gln188Arg variant has no effect towards the metal binding capacity of hGALT, as shown by ITC (Fig. 5B and C) and DSF (Supplementary Material, Fig. S9B).

### Zinc and uridylylation alter aggregation kinetics of hGALT

Recently an increased tendency to aggregate was reported for the p.Gln188Arg variant (31). In light of our structural findings, we further investigated p.Gln188Arg aggregation by studying the effect of uridylyation and zinc binding on turbidity (Fig. 5D). As observed before, we found as-purified apo-hGALT, UMP-hGALT and hGALT(p.Q188R) to have a tendency to aggregate at 37 °C. hGALT(p.Q188R) is more prone to aggregation than UMP-hGALT, but shows similar aggregation rates to that of apo-hGALT. This suggests that uridylylation slows down the kinetics of hGALT aggregation, and thus the aggregation of hGALT(p.Q188R) is likely associated with its reduced level of uridylylation. Strikingly, excess Zn^2+ ^prevented aggregation (Fig. 5D, green coloured line) and excess ethylenediaminetetraacetic acid (EDTA) slightly increased the rate of aggregation (Fig. 5D, purple coloured line) for all hGALT species during the course of the experiment. The Aggrescan server (36) predicted that the hGALT active site, with a β-strand rich topology, is a hot spot for aggregation (Supplementary Material, Fig. S10A and B), which could potentially be altered by uridylylation at the active site His186. Additionally, the hGALT-bound Zn^2+ ^ion is linked to the active site ∼20 Å away via strand β7 and helix α2 along with the Zn^2+^-coordinating residue Glu201 (Supplementary Material, Fig. S10C). Our results suggest a higher tendency of hGALT(p.Q188R) to aggregate due to its reduced ability to be uridylylated, and confirm that Zn^2+ ^acts as a structurally important ion. Further studies are warranted to determine the aggregation tendency of other variants where uridylylation is not affected.

## Discussion

Our 1.9 Å crystal structure of the hGALT ternary complex offers an experimentally determined structural explanation for the majority of galactosemia-associated mutations in the context of bound divalent metal ions and dimerisation interactions. This expands on and improves the existing understanding of their effects largely derived from previous homology models of hGALT (28,31,37). Importantly, our structure clarifies the ambiguity on the number of metal binding sites and agrees with previous observation of enzyme-bound zinc (27). In addition, we have shown that metal binding and uridylylation conferred effects to enzyme stability with consequences for disease-associated mutants. Since the original report of the eGALT structure, it has been apparent that this enzyme is quite heterogeneous in terms of its ligands (23–25), although no work on their effects towards enzyme stability has been reported. It is of note that various cofactors and modifications can alter a metabolic enzyme’s turnover and stability, with functional association to disease (38). As renewed interest in the mechanistic basis of galactosemia has shown that disease-associated mutants exert their effects on misfolding through affecting expression (39), solubility (33,40), stability (31,40,41) and aggregation tendency (31) of hGALT, it is critical to understand the contribution of these factors. The previous lack of consideration of the effects of covalent modification by UMP and divalent metal binding has been in part due to the absence of a crystal structure of the human protein.

The crystal structure and biophysical studies presented here answer these questions by showing that the covalent modification at His186 by UMP has structural effects on hGALT. This is previously unrecognised for GALT enzymes, but observed for other covalent modifications such as pyridoxal 5′-phosphate for alanine: glyoxylate aminotransferase (42) and phosphorylation of phosphoglucomutase (43). These factors play a large role in the stability and activity of their counterpart enzymes. Our intact mass spectrometry analysis extends previous observations using two-dimensional electrophoresis (32), to quantitatively show that p.Q188R has a reduced ability to become uridylylated. The previous study showed that hGALT is present as both apo and covalently modified species *in vivo* whereas an active site variant (p.His186Gly, similar to our p.His186Ala variant) is only present in the apo form. Our findings further suggest that p.Gln188Arg will be present mostly as the apo species *in vivo*, and thus may be more susceptible to aggregation and degradation. Certain mutations may give rise to hGALT with lower activity, making it more susceptible to degradation, which would explain the findings of low hGALT activity in only the peripheral tissues of some patients homozygous for the p.S135L variant (34).

Importantly, we find that the single divalent metal binding site of hGALT is stabilising in nature, which has its parallels in superoxide dismutase and p53, involved in amyotrophic lateral sclerosis (44) and cancer (45), respectively. Our studies show that different metal ions can potentially bind at this site but it is unclear if hGALT is present in different metallated states *in vivo*, although the preference appears to be Zn^2+ ^(27). Zinc homeostasis is poorly understood and its concentration varies across different cellular compartments but falls on average in the micromolar range (46) similar to our determined *K_d_* for hGALT. Our studies demonstrate that stability is linked to the availability of zinc and other divalent metal ions, and zinc deficiency may exasperate the effects of many hGALT variants (47). Therefore, zinc supplementation may be beneficial as treatment in some cases of galactosemia, as it is possible that amino acid substitutions distant from this site may alter its binding and affinity. Further studies are warranted to determine if altered metal binding is a common feature of classic galactosemia, although this appears not to be the case for p.Gln188Arg. Interestingly, to the best of our knowledge, there have been no reports of aggregation of hGALT *in vivo* or a toxic gain of function.

Our structure will also guide future developments of therapies for the disease. Focus has been on substrate reduction therapies by targeting GALK1 to decrease galactose-1-phosphate concentrations (48), and recently superoxide dismutase mimics appear to treat a fruit fly model of galactosemia (49). Another therapeutic approach would be to increase the activity of hGALT, either by enzyme replacement therapy or by small-molecule chemical/pharmacological chaperones (31,40,50,51). The latter is the more economical option based on the experience of lysosomal storage disorders (52). In support of a chaperone approach, the chemical chaperone arginine has been reported to rescue bacteria expressing the p.Gln188Arg and p.Lys285Asn variants (53). For hGALT-specific pharmacological chaperones, the dimer interface on opposite face of the active sites (40) or the divalent metal binding sites are potential pockets to be targeted for small molecule design and screening, akin to the efforts in amyotrophic lateral sclerosis (54) and transthyretin amyloidoses (55). Overall, our findings provide a comprehensive basis for understanding the molecular consequences of hGALT mutations at the protein level, and will guide future efforts in developing chemical and pharmacological chaperones as alternative therapeutics for classic galactosemia.

## Materials and Methods

### Cloning, expression and purification of hGALT

DNA fragment encoding full-length hGALT was amplified from a cDNA clone that harbours the p.Asn314Asp polymorphism (11) (IMAGE: 3922902), and subcloned into the pNIC28-Bsa4 vector that incorporates an N-terminal TEV-cleavable His6-tag. hGALT variants were created using the Quik-Change method in the context of our cDNA clone carrying the p.Asn314Asp polymorphism. All hGALT variants were subjected to the following purification procedure: hGALT was cultured in 6 l of Terrific Broth at 37 °C, and induced with 0.1 mm IPTG overnight at 18 °C. Cell pellets were harvested, homogenized in lysis buffer (50 mm sodium phosphate pH 7.5, 500 mm NaCl, 5% glycerol, 0.5 mm TCEP) and centrifuged to remove insoluble material. The supernatant was purified by affinity (Talon resin; GE Healthcare) and size-exclusion (Superdex 200; GE Healthcare) chromatography. Purified protein was treated with His-tagged TEV protease overnight at 4 °C, and further purified by reverse Nickel affinity and size exclusion chromatography, concentrated to 20 mg∕ml and stored in storage buffer (50 mm HEPES pH 7.5, 500 mm NaCl, 5% glycerol, 0.5 mm TCEP) at −80 °C. To produce apo and uridylylated protein, tag-removed hGALT (10 mg/ml) was incubated overnight at 4 °C with 2 mm glucose 1-phosphate or UDP-glucose before the final size-exclusion step. Samples were analysed by denaturing mass spectrometry.

### *In vitro* de-uridylylation and uridylylation assay

20 µm purified hGALT was incubated in 50 mm Na-citrate pH 5.5, 150 mm NaCl, 5% (v∕v) glycerol, 0.5 mm TCEP with either 2 mm glucose 1-phosphate or UDP-glucose at 4 °C for 12 h. Protein intact mass was determined using an MSD-TOF electrospray ionization orthogonal time-of-flight mass spectrometer and analysed by TOF Protein Confirmation Software (Agilent).

### Crystallization, data collection and structure determination

Crystals were grown by vapour diffusion at 20 °C, from sitting drops mixing 200 nl of protein (20 mg/ml; pre-incubated with 5 mm UDP-Glc) and 100 nl of reservoir solution containing 0.2 M ammonium sulphate, 30% (w/v) PEG 8000. Crystals were cryo-protected with reservoir solution supplemented with 25% (v/v) ethylene glycol and flash-cooled in liquid nitrogen. Diffraction data were collected on the Diamond Light Source beamlines and processed using the CCP4 Suite (56). The structure was solved by molecular replacement with PHASER (57), using the structure of eGALT (1HXP) as template. Modelling and refinement were carried out using Refmac (58) and Coot (59). The asymmetric unit contains two polypeptide chains and difference maps clearly showed the presence of UMP covalently bound to His186 along with one glucose-1-phosphate molecule ion and one zinc ion per monomer. There was no interpretable electron density for residues 1–27, 49–63, 367–379 in chain A and residues 1–23, 49–63, 365–379 in chain B. Dimer interactions was analysed by using the PISA server (http://www.ebi.ac.uk/pdbe/pisa/).

### High pressure liquid chromatography single angle X-ray scattering

Protein was subjected to high pressure liquid chromatography single angle X-ray scattering at 10 mg/ml in 10 mm HEPES, pH 7.5, 150 mm NaCl, 0.5 mm TCEP, by flowing sample through an in-vacuum quartz capillary of 1.6 mm diameter. Data were collected using a Pilatus2M detector (Dectris, CH) at a sample-detector distance of 3914 mm and a wavelength of λ = 1 Å. The range of momentum transfer of 0.1 < s < 5 nm^−^^1^ was covered (*s* = 4πsinθ/λ, where θ is the scattering angle). Batch mode was used where a comparison of eighteen 10s exposures was performed. Radiation damage was monitored by observing changes in the radius of gyration in each frame, where no significant changes were observed. The data were radially averaged and the scattering of the buffer was subtracted. The forward scattering *I*(0), radius of gyration *R*_g_, pair distribution of the particle *P*(r) and maximum dimension *D*_max_ were analysed using ScÅtter (http://www.bioisis.net) and the ATSAS suite of programs (60).

### Differential scanning fluorimetry

DSF was performed in a 96-well plate using an Mx3005p RT-PCR machine (Stratagene) with excitation and emission filters of 492 and 610 nm, respectively. Each well (20 µl) consisted of protein (2 µm in buffer containing 50 mm Na-citrate, pH 5.5, and 500 mm NaCl), SYPRO-Orange (Invitrogen, diluted 1000-fold of the manufacturer’s stock), and various concentrations of ligand. Fluorescence intensities were measured from 25 to 96 °C with a ramp rate of 1 °C/min. *T*_m_ was determined by plotting the intensity as a function of temperature and fitting the curve to a Boltzmann equation (63). Temperature shifts, Δ*T*_m_, were determined as described (61) and final graphs were generated using GraphPad Prism (v.5.01; Graph-Pad Software). Assays were carried out in triplicate.

### Native proteolysis

Native proteolysis with proteinase K was carried out in 50 mm Na-citrate, pH 5.5, 500 mm NaCl at 20 °C (62). Remaining intact protein for different time points was determined by a combination of sodium dodecyl sulphate–polyacrylamide gel electrophoresis and ImageJ software (http://rsbweb.nih.gov/ij/), the latter of which was used to determine band intensities. Rates of proteolysis (*k_p_*) were determined by plotting the percentage of remaining intact protein against time and fitting to an exponential decay function. ZnCl_2_ was added at a final concentration of 100 µM when appropriate. Assays were carried out in triplicate.

### Isothermal titration calorimetry

Protein samples were buffer exchanged to 10 mm Na-citrate, pH 5.5, 200 mm NaCl using PD-10 columns (GE Healthcare), flash frozen in liquid nitrogen and stored at − 80 °C. ZnCl_2_ was dissolved in water at 100 mm and diluted in 10 mm Na-citrate, pH 5.5, 200 mm NaCl. ITC experiments were carried out in an ITC_200_ machine (Malvern Instruments). Proteins were loaded into the calorimetric cell at 17–20 μm (in monomer) and ZnCl_2_ was loaded into the titrating syringe at 650 μm. Titrations were performed at 25°C. After an initial injection of 0.5 μl, 25 injections of 1.5 μl were performed, separated by 2.5 min. The enthalpograms corresponding to GALT samples were corrected for heats of dilution using a blank titration without protein. The binding isotherms were analysed using a single type of identical and independent binding sites, and the number of sites was fixed to 1:1 (per monomer) due to the low binding affinity.

### Aggregation assay

20 μm purified hGALT was incubated in 50 mm Na-citrate pH 5.5, 150 mm NaCl, 5% (v/v) glycerol, 0.5 mm TCEP at 37 °C for 150 min in total volume of 200 µl. In some cases EDTA or ZnCl_2_ was added at 1 mm. Propensity to aggregate was measured over time at an absorbance of 350 nm as an indicator of turbidity. Assays were carried out in triplicate.

## Supplementary Material

Supplementary Material is available at *HMG* online.

Supplementary Data
