# Supplementary material for: Molecular basis of classic galactosemia from the structure of human galactose 1-phosphate uridylyltransferase
Source: Hum Mol Genet. 2016 Mar 22;25(11):2234–44. doi: 10.1093/hmg/ddw091 (PMC5081055; doi:10.1093/hmg/ddw091)
Supplement: Supplementary Data [file supp_25_11_2234__index.html]

Molecular basis of classic galactosemia from the structure of human galactose 1-phosphate uridylyltransferase — Molecular basis of classic galactosemia from the structure of human galactose 1-phosphate uridylyltransferase — Supplementary Data 

# Molecular basis of classic galactosemia from the structure of human galactose 1-phosphate uridylyltransferase

## Supplementary Data

files

- Supplementary Data - pdf file
