# Supplementary material for: Molecular basis of classic galactosemia from the structure of human galactose 1-phosphate uridylyltransferase
Source: Hum Mol Genet. 2016 Mar 22;25(11):2234–44. doi: 10.1093/hmg/ddw091 (PMC5081055; doi:10.1093/hmg/ddw091)
Supplement: Supplementary Data [file supp_ddw091_SupplementaryInformation.pdf]

## SUPPLEMENTARY INFORMATION

# Molecular basis of classic galactosemia from the structure of human galactose 1-phosphate uridylyltransferase.

Thomas J. McCorvie[a], Jolanta Kopec[a], Angel L. Pey[b], Fiona Fitzpatrick[a,c], Dipali Patel[a], Rod Chalk[a], Leela Streetha[a], Wyatt W. Yue[a,1]

[a]Structural Genomics Consortium, Nuffield Department of Clinical Medicine, University of Oxford, UK OX3 7DQ

[b]Department of Physical Chemistry, Faculty of Sciences, University of Granada, Spain, E-18071

[c]Present address: University of Cambridge, MRC Mitochondrial Biology Unit, Wellcome Trust/MRC
Building, Hills Road, Cambridge, CB2 0XY

[1]To whom correspondence should be addressed:
W.W.Y. (wyatt.yue@sgc.ox.ac.uk),

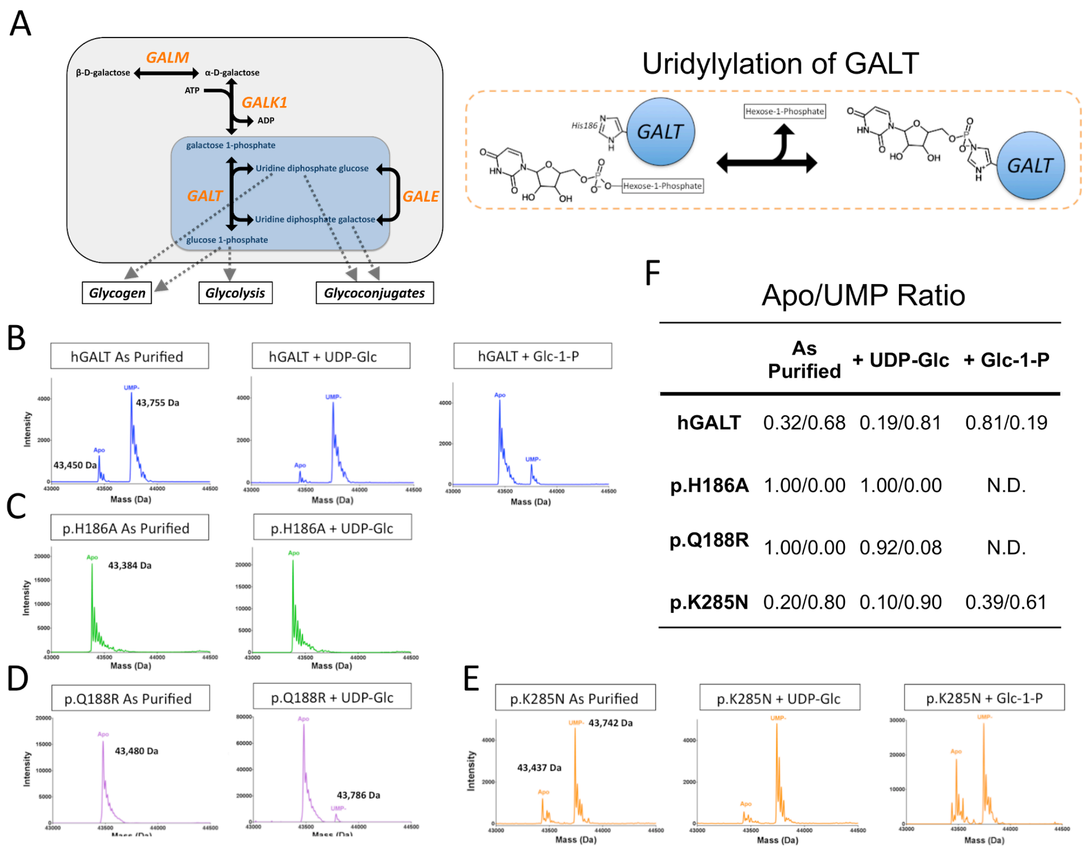


| | As Purified | + UDP-Glc | + Glc-1-P |
|---|---|---|---|
| hGALT | 0.32/0.68 | 0.19/0.81 | 0.81/0.19 |
| p.H186A | 1.00/0.00 | 1.00/0.00 | N.D. |
| p.Q188R | 1.00/0.00 | 0.92/0.08 | N.D. |
| p.K285N | 0.20/0.80 | 0.10/0.90 | 0.39/0.61 |

**Supplementary Figure 1 | Uridylation of hGALT. (A)** The Leloir pathway consists of four enzymes, which are shown in orange boldface. The GALT enzyme reaction is highlighted blue. The metabolites of the Leloir pathway are used for three main functions: the formation of glycogen as energy storage; usage in glycolysis to produce ATP; and in the creation of glycoconjugates (glycolipids and glycoproteins). hGALT carries out its enzymatic reaction with a covalent intermediate: Here the UMP group of the UDP-hexose substrate is reversibly attached to active site His186 resulting in the release of the hexose-1-phosphate. **(B)** Intact denaturing mass spectrometry of purified hGALT shows the presence of both an apo and uridylylated species. The ratios of these species are altered by incubation with either UDP-Glc or Glc-1-P. **(C)** Mutation of the active site histidine to an alanine results in only one species (apo) being detected, which shows no modification by UMP when incubated with UDP-Glc. **(D)** The most common classic galactosemia-associated variant, p.Q188R, is also purified mostly in the apo form. Incubation with UDP-Glc overnight clearly shows the presence of uridylylated protein, however this of considerably lower signal than that obtained with hGALT showing this variant has a very low activity. **(E)** Another common variant, p.K285N, though only purified to a low yield and quality, clearly shows the presence of two species corresponding to the apo and uridylylated variant protein. The ratio of these species is altered by incubating with substrates and shows this variant to be significantly

active. **(F)** Table of corresponding ratios of apo vs UMP for each hGALT protein and incubation experiment.

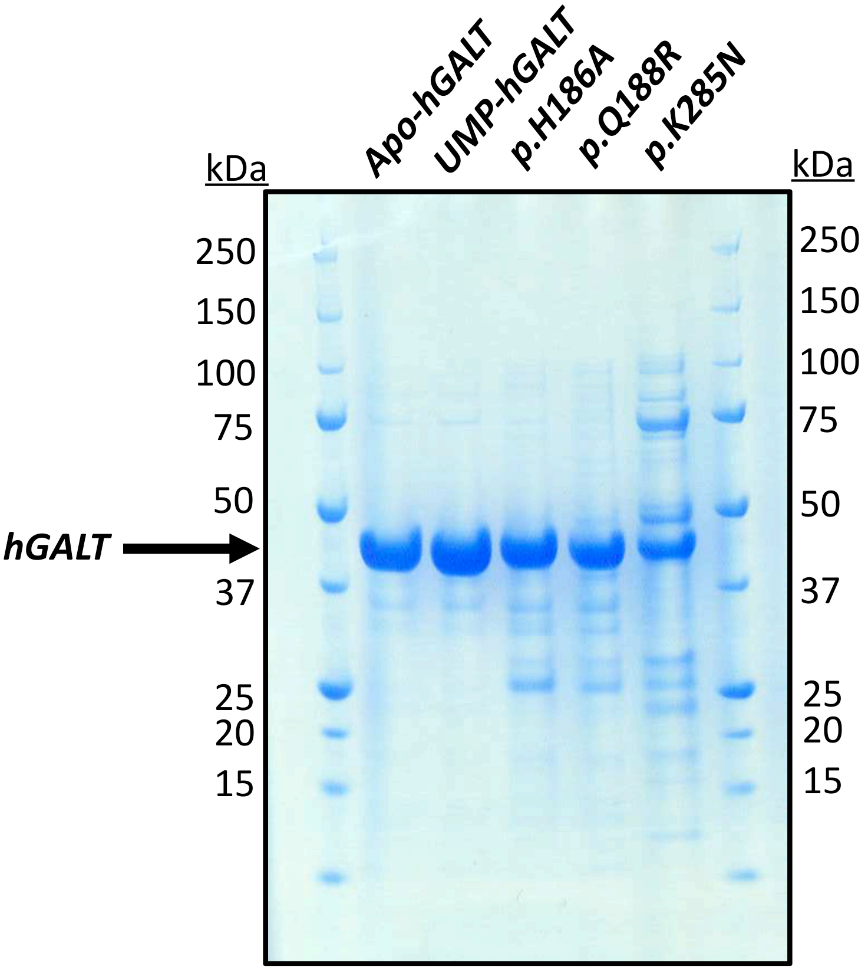


**Supplementary Figure 2 | Coomassie stained SDS-PAGE of the hGALT proteins in this study.** Apo-hGALT and UMP-hGALT proteins were purified to greater than yield of 90%. The variant proteins p.H186A and p.Q188R were purified to a yield of ≈ 90%. The yield and quality of p.K285N was low due to the poor expression of this variant (≈ 3 mg/L for apo-hGALT vs ≈ 0.2 mg/L for p.K285N).

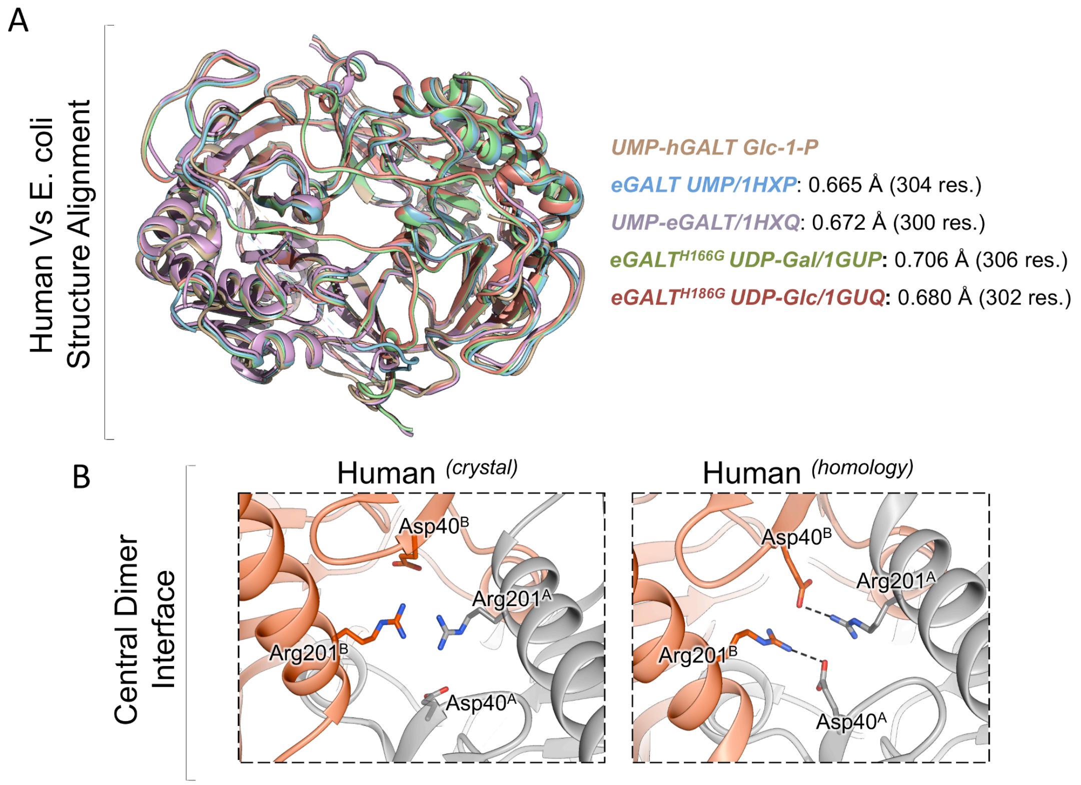


**Supplementary Figure 3 | Comparison of our hGALT crystal structure against other GALT structures. (A)** Structural alignment of the hGALT crystal structure against the various structure of eGALT. The average $C^{\alpha}$-RMSD value is ≈ 0.7 Å **(B)** Comparison of the salt-bridge interactions predicted by the hGALT homology model. The only predicted salt-bridge is not present in our crystal structure due to the orientation of Asp40 in both chains. This residue in both chains points towards the centre of the protein, preventing interaction with Arg201.

Agrees

## Crystal Structure

| ## | Chain B | Dist. [Å] | Chain A |
|---|---|---|---|
| 1 | B:GLN 103[ N ] | 2.95 | A:GLN 30[ OE1] |
| 2 | B:LYS 120[ N ] | 2.91 | A:ILE 32[ O ] |
| 3 | B:ARG 48[ NH2] | 2.80 | A:PHE 99[ O ] |
| 4 | B:ARG 48[ NE ] | 3.50 | A:PRO 100[ O ] |
| 5 | B:SER 45[ OG ] | 2.81 | A:ALA 101[ O ] |
| 6 | B:HIS 47[ NE2] | 2.86 | A:ALA 101[ O ] |
| 7 | B:GLN 30[ NE2] | 2.77 | A:GLN 103[ O ] |
| 8 | B:ARG 228[ NH2] | 2.88 | A:ASP 113[ OD1] |
| 9 | B:TYR 34[ N ] | 3.07 | A:GLN 118[ O ] |
| 10 | B:ILE 32[ N ] | 2.97 | A:LYS 120[ O ] |
| 11 | B:GLN 30[ NE2] | 2.95 | A:ALA 122[ O ] |
| 12 | B:TRP 41[ N ] | 3.01 | A:ASP 197[ OD1] |
| 13 | B:HIS 114[ NE2] | 2.76 | A:GLU 220[ OE2] |
| 14 | B:GLY 338[ N ] | 3.04 | A:SER 297[ OG ] |
| 15 | B:HIS 301[ NE2] | 2.56 | A:LEU 342[ O ] |
| 16 | B:ASP 197[ N ] | 3.46 | A:ALA 343[ O ] |
| 17 | B:ILE 198[ N ] | 2.79 | A:ALA 343[ O ] |
| 18 | B:ASP 197[ N ] | 3.05 | A:GLN 344[ OE1] |
| 19 | B:GLN 30[ OE1] | 2.84 | A:GLN 103[ N ] |
| 20 | B:ILE 32[ O ] | 2.94 | A:LYS 120[ N ] |
| 21 | B:ALA 101[ O ] | 2.79 | A:SER 45[ OG ] |
| 22 | B:GLN 103[ O ] | 2.80 | A:GLN 30[ NE2] |
| 23 | B:ASP 113[ OD1] | 3.22 | A:ARG 228[ NH2] |
| 24 | B:ASP 113[ OD2] | 3.38 | A:ARG 228[ NH2] |
| 25 | B:GLN 118[ O ] | 2.96 | A:TYR 34[ N ] |
| 26 | B:LYS 120[ O ] | 2.94 | A:ILE 32[ N ] |
| 27 | B:ALA 122[ O ] | 2.92 | A:GLN 30[ NE2] |
| 28 | B:ASP 197[ OD1] | 3.10 | A:TRP 41[ N ] |
| 29 | B:GLU 220[ OE2] | 2.83 | A:HIS 114[ NE2] |
| 30 | B:SER 297[ OG ] | 2.97 | A:GLY 338[ N ] |
| 31 | B:LEU 342[ O ] | 2.64 | A:HIS 301[ NE2] |
| 32 | B:ALA 343[ O ] | 2.88 | A:ILE 198[ N ] |
| 33 | B:ALA 343[ O ] | 3.34 | A:ASP 197[ N ] |
| 34 | B:GLN 344[ OE1] | 2.75 | A:ASP 197[ N ] |

## Homology Model

| ## | Chain A | Dist. [Å] | Chain B |
|---|---|---|---|
| 1 | A:ALA 122[ N ] | 3.65 | B:GLN 30[ O ] |
| 2 | A:LYS 120[ N ] | 2.99 | B:ILE 32[ O ] |
| 3 | A:ARG 201[ NH2] | 3.00 | B:GLU 40[ OE2] |
| 4 | A:ASN 182[ ND2] | 2.99 | B:GLN 54[ OE1] |
| 5 | A:GLN 56[ NE2] | 3.70 | B:GLU 58[ OE1] |
| 6 | A:GLN 30[ NE2] | 3.41 | B:PRO 100[ O ] |
| 7 | A:SER 45[ OG ] | 3.15 | B:ALA 101[ O ] |
| 8 | A:TYR 34[ N ] | 3.43 | B:GLN 118[ O ] |
| 9 | A:ILE 32[ N ] | 3.01 | B:LYS 120[ O ] |
| 10 | A:GLN 54[ NE2] | 3.01 | B:SER 181[ O ] |
| 11 | A:GLN 54[ N ] | 3.25 | B:SER 181[ O ] |
| 12 | A:TRP 41[ N ] | 2.99 | B:ASP 197[ OD1] |
| 13 | A:HIS 301[ NE2] | 2.80 | B:LEU 342[ O ] |
| 14 | A:ASP 197[ N ] | 3.40 | B:ALA 343[ O ] |
| 15 | A:ILE 198[ N ] | 3.21 | B:ALA 343[ O ] |
| 16 | A:ASP 197[ N ] | 3.16 | B:GLN 344[ OE1] |
| 17 | A:GLN 30[ O ] | 3.83 | B:ALA 122[ N ] |
| 18 | A:ILE 32[ O ] | 3.04 | B:LYS 120[ N ] |
| 19 | A:GLU 40[ OE2] | 2.99 | B:ARG 201[ NH2] |
| 20 | A:GLN 54[ OE1] | 2.98 | B:ASN 182[ ND2] |
| 21 | A:GLU 58[ OE1] | 3.65 | B:GLN 56[ NE2] |
| 22 | A:PRO 100[ O ] | 3.35 | B:GLN 30[ NE2] |
| 23 | A:ALA 101[ O ] | 3.04 | B:SER 45[ OG ] |
| 24 | A:GLN 118[ O ] | 3.34 | B:TYR 34[ N ] |
| 25 | A:LYS 120[ O ] | 2.97 | B:ILE 32[ N ] |
| 26 | A:GLN 169[ OE1] | 3.84 | B:ALA 343[ N ] |
| 27 | A:SER 181[ O ] | 3.20 | B:GLN 54[ N ] |
| 28 | A:SER 181[ O ] | 3.00 | B:GLN 54[ NE2] |
| 29 | A:SER 181[ OG ] | 3.80 | B:ARG 51[ NE ] |
| 30 | A:ASP 197[ OD1] | 2.95 | B:TRP 41[ N ] |
| 31 | A:LEU 342[ O ] | 2.83 | B:HIS 301[ NE2] |
| 32 | A:ALA 343[ O ] | 3.27 | B:ILE 198[ N ] |
| 33 | A:ALA 343[ O ] | 3.40 | B:ASP 197[ N ] |
| 34 | A:GLN 344[ OE1] | 3.22 | B:ASP 197[ N ] |

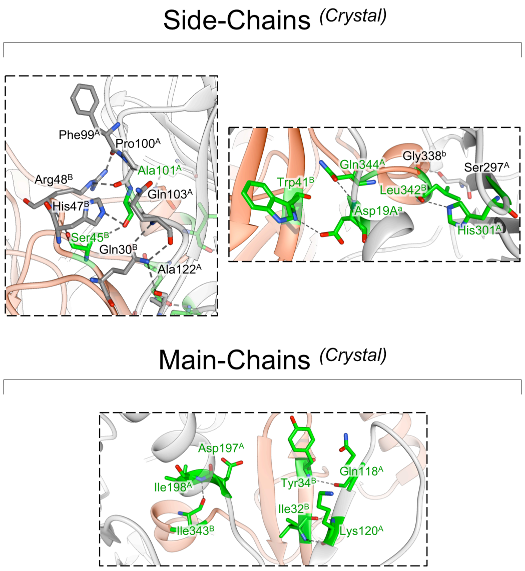


**Supplementary Figure 4 | Comparison of the dimer interactions for our crystal structure and the homology model.** Dimer interactions were analysed using the PISA server and are listed in both tables. Correctly predicted interactions are highlighted green. Dimer interactions in the hGALT crystal structure are shown for both side-chain and main chain interactions. Residues involved are depicted as sticks and those predicted to interact by the homology model are coloured green. This shows that the homology model does not accurately predict a number of side-chain interactions.

A B

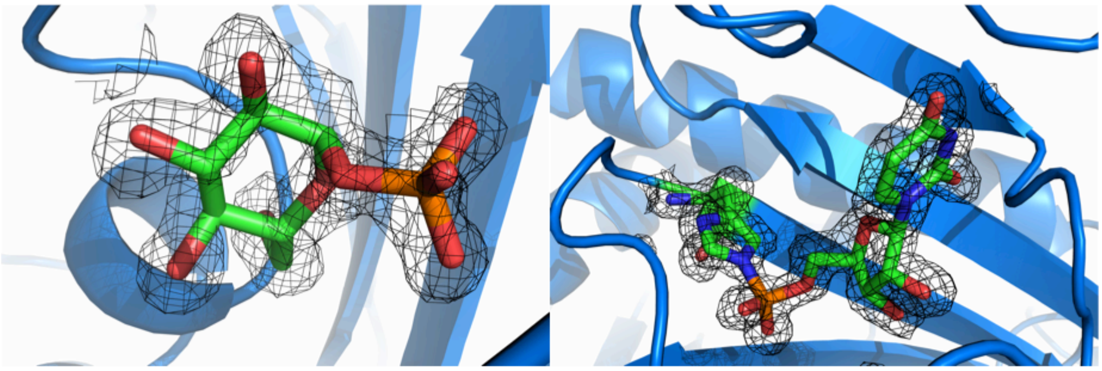

**Supplementary Figure 5 | Feature enhanced maps showing the ligands present within the active site of our 1.9 Å structure of hGALT. (A)** Glc-1-P within the active site of chain A. **(B)** Covalent modification by UMP of His186 at the active site of chain A.

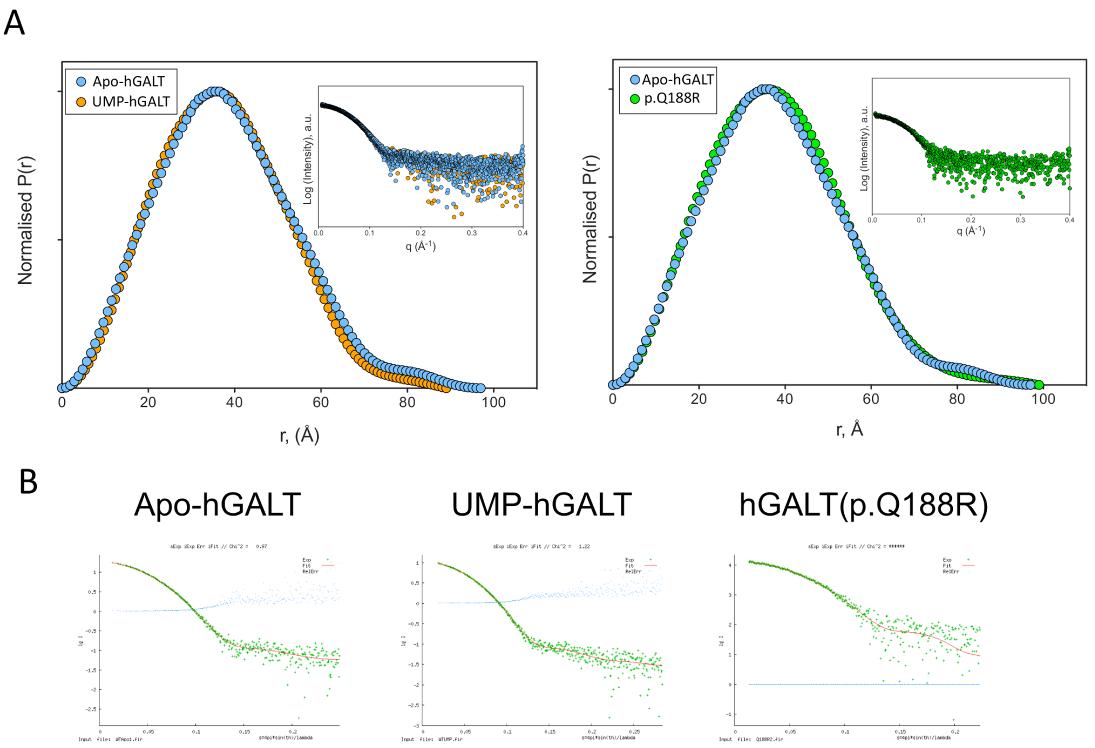


**Supplementary Figure 6 | SAXS analysis of apo-hGALT, UMP-hGALT, and hGALT(p.Q188R).** **(A)** Calculated *P(r)* curves of apo versus UMP-hGALT and apo-hGALT versus hGALT(p.Q188R) as determined by ScÅtter. These show that hGALT is a global protein in solution, that uridylylation causes a contraction and that hGALT(p.Q188R) is slightly larger than apo-hGALT. Insets are the HPLC-SAXS intensity curves of the proteins. **(B)** SAXS intensity plots with the simulated plots of the models as determined by GASBOR.

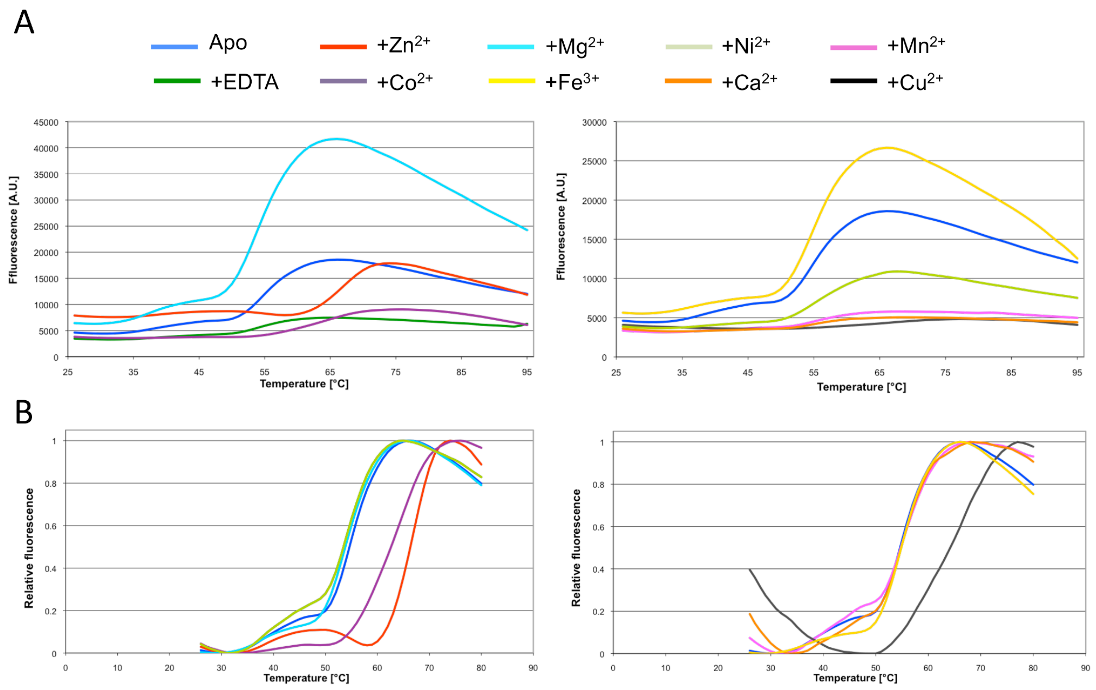


**Supplementary Figure 7 | DSF screening of hGALT metal binding.** Apo-hGALT at 2 µM was used to screen against EDTA and various metal ions at 0.5 mM. **(A)** Representative unfolding curves. **(B)** Corresponding normalised unfolding curves. Apo-hGALT shows biphasic unfolding in all conditions tested except with $Cu^{2+}$ where the initial low temperature transition is not present. This is likely due to the low signal of unfolding in comparison to other conditions tested. Divalent metal binding appears to only affect the higher temperature transition.

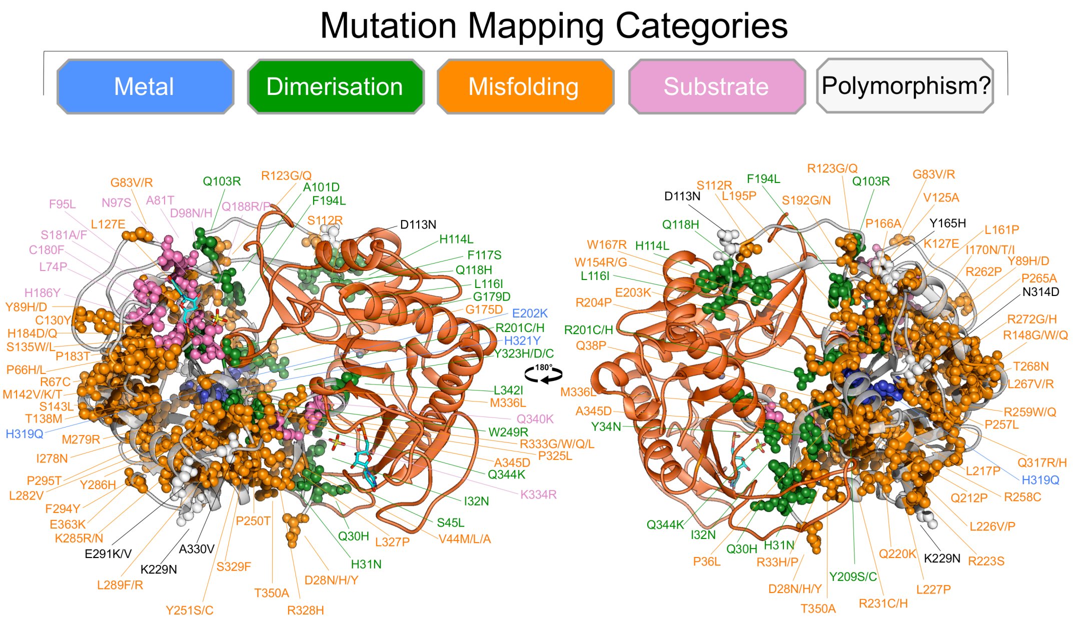


**Supplementary Figure 8 | Structural mapping and categorisation of the missense mutations found in the hGALT gene.** Missense mutations listed in the GALT mutational database (http://arup.utah.edu/database/GALT/GALT_display.php) were mapped on the hGALT dimer and are depicted as sticks and balls. Each mutation was categorised depending on its predicted effect on the protein crystal structure. Blue: metal binding; Green: dimerisation; Orange: misfolding; Pink: substrate binding; Black: polymorphism or unknown effect.

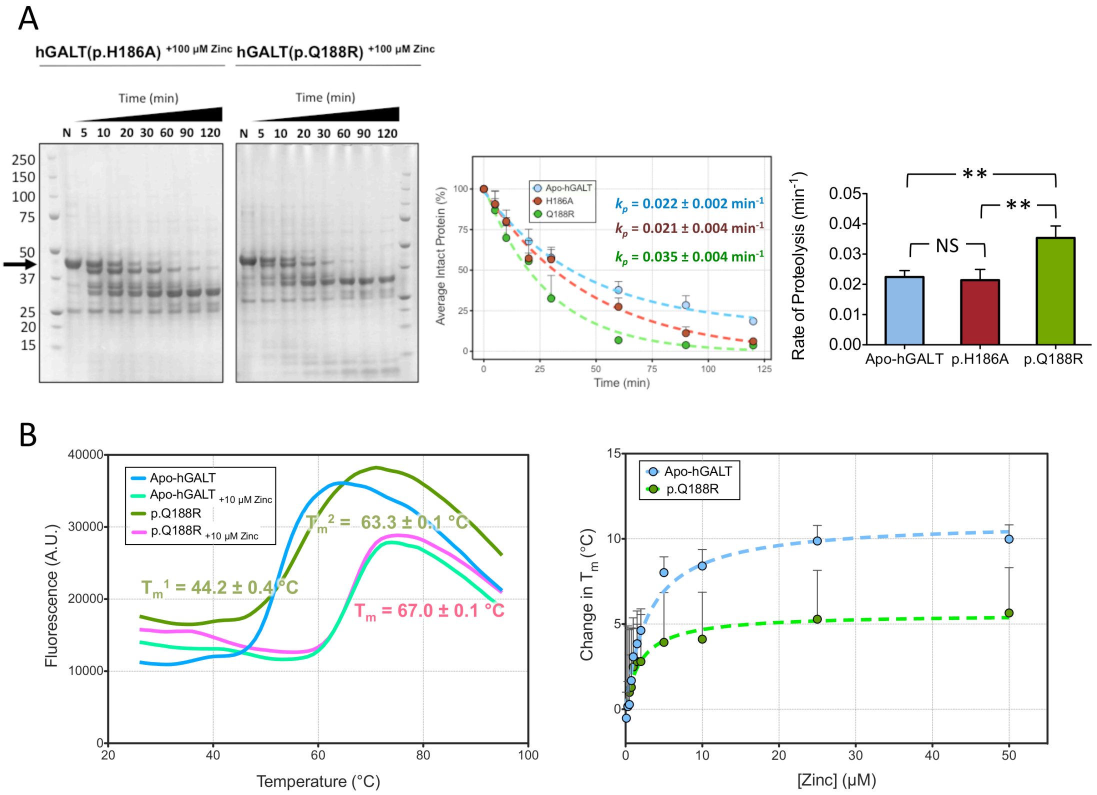


**Supplementary Figure 9 | Biophysical consequences of the p.Q188R variant. (A)** Representative native proteolysis SDS-PAGE gels of hGALT(p.H186A) and hGALT(p.Q188R) in the presence of excess zinc. The determined rates shows that hGALT(p.Q188R) is slightly more susceptible to proteolysis than both apo-hGALT and hGALT(p.H186A) indicative of a misfolding effect. Rates of proteolysis are reported as the means and standard deviations from at least three independent experiments. *p* values were determined using two-tailed unpaired t test. NS: non-significant; **: $p < 0.01$. **(B)** DSF analysis of hGALT(p.Q188R) vs apo-hGALT showing representative unfolding curves. As purified, hGALT(p.Q188R) ($T_m^1$ = 44.2 °C, $T_m^2$ = 63.3 °C) demonstrated a biphasic-unfolding curve with higher melting temperatures than apo-hGALT ($T_m^1$ = 39.6 °C, $T_m^2$ = 54.5 °C). Though the first transition was of low signal this was routinely detected in all replicates for hGALT(p.Q188R). In the presence of excess zinc both proteins were stabilised to a similar stability with apo-hGALT: $T_m^2$ = 66.9 °C; and hGALT(Q188R): $T_m^2$ = 67.0 °C. Annotated melting temperatures are for hGALT(p.Q188R) only. Please see Figure 2 for apo-hGALT values. Dose response curves showed a lower change in melting temperature for Q188R due to its higher basal stability.

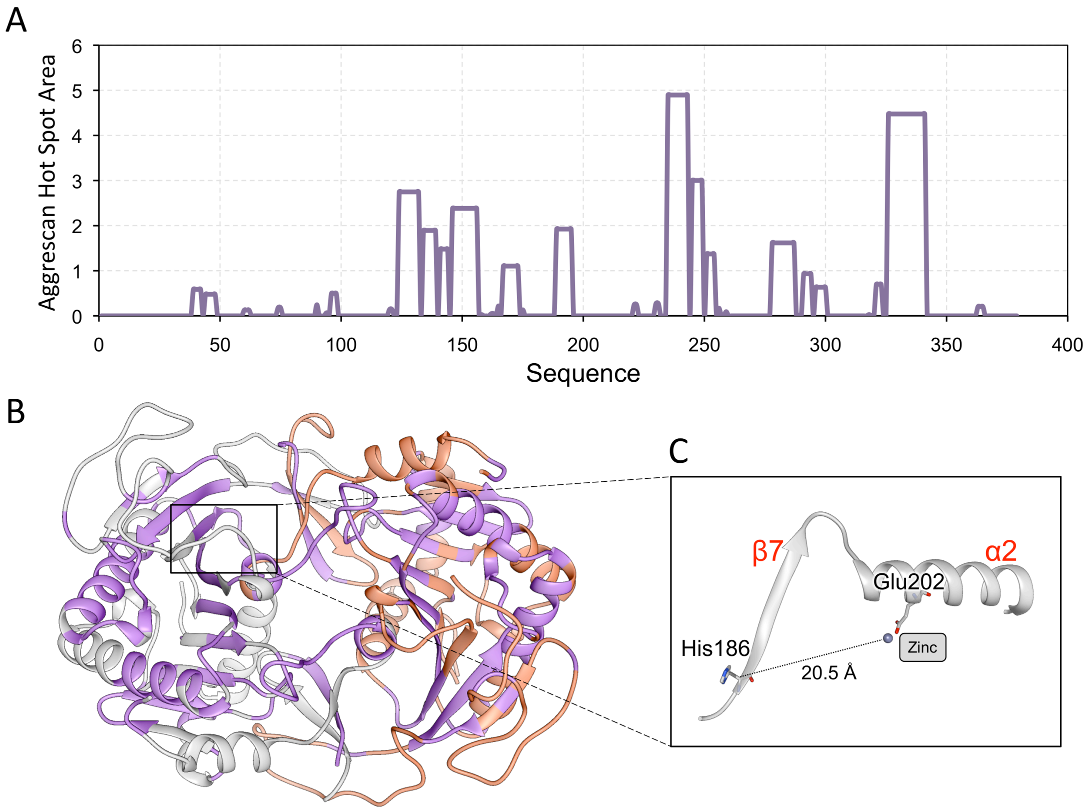


**Supplementary Figure 10| Predicted aggregation propensity of hGALT.** (A) Predicted regions of aggregation in hGALT determined by the Aggrescan server (http://bioinf.uab.es/aggrescan/) (B) Mapping of the predicted regions on the hGALT dimer (purple colouring) demonstrate the active site, metal binding site and surrounding area are potentially prone to aggregation. (C) Though ≈ 20 Å away, the zinc-binding site is connected to the active site through Glu202 on α2 and β7. The metal binding site likely stabilises the entire extended β-sheet structure of hGALT.

**Supplementary Table 1 | Crystallography refinement statistics.**

| | **hGALT** |
|---|---|
| PDB Code | 5IN3 |
| **Data collection and processing** | |
| Beamline | I02 |
| Wavelength | 0.97949 |
| Unit cell parameters (Å) | 59.8 108.0 126.4 |
| (°) | 90 90 90 |
| Space group | $P2_1\ 2_1\ 2_1$ |
| Resolution range (Å) | 54.99-1.90 (1.94-1.90) |
| Observed/Unique reflections | 624060/66370 (40320/4211) |
| Rsym(%) | 15.4 (115) |
| CC(1/2) | 0.996 (0.654) |
| I/sig(I) | 11.0 (1.5) |
| Completeness | 99.8 (99.4) |
| Multiplicity | 9.4 (9.6) |
| Wilson *B* factor (Å$^2$) | 27.68 |
| **Refinement** | |
| Rwork (%) | 19.8 |
| Rfree (%) | 22.8 |
| Average total *B* factor (Å$^2$) | 35.2 |
| Average ligand *B* factor (Å$^2$) | 54.1 |
| Ligand occupancy | UMP = 1<br>G1P = 0.7 |
| R.m.s.d. bond length (Å) | 0.009 |
| R.m.s.d. bond angle (°) | 1.06 |
| **Molprobity analysis** | |
| Clashscore | 3.52 |
| Ramachadran favoured (%) | 97 |
| Ramachandran outliers (%) | 0 |
| Rotamer outliers (%) | 1 |

Data for highest resolution shell are shown in parenthesis.

**Supplementary Table 2 | Predicted structural consequences of hGALT variants**

| No. | Nucleotide Change | Protein Change | Effect | Category |
|---|---|---|---|---|
| *1* | c.1A>G | p.M1V | Disordered N-terminus, delayed translation | N/O |
| *2* | c.27G>C | p.Q9H | Disordered N-terminus, unknown | N/O |
| *3* | c.67A>G | p.T23A | Disordered N-terminus, unknown | N/O |
| *4* | c.82G>A | p.D28N | Remove H-bond with Arg25 | Misfolding |
| *5* | c.82G>C | p.D28H | Remove H-bond with Arg25 | Misfolding |
| *6* | c.82G>T | p.D28Y | Remove H-bond with Arg25 | Misfolding |
| *7* | c.90G>C | p.Q30H | Remove inter-chain H-bond with Gn103, Pro104 and Ala122 | Dimerisation |
| *8* | c.91C>A | p.H31N | Remove H-bond with Arg33 | Dimerisation |
| *9* | c.95T>A | p.I32N | Steric clash with Gln344 | Dimerisation |
| *10* | c.98G>A | p.R33H | Remove H-bonds with Phe245 and Glu352 | Misfolding |
| *11* | c.98G>C | p.R33P | Remove H-bonds with Phe245 and Glu352, decrease flexibility | Misfolding |
| *12* | c.100T>A | p.Y34N | Remove H-bond with Arg39 | Dimerisation |
| *13* | c.107C>T | p.P36L | Steric clash with Leu116 of interacting chain | Misfolding |
| *14* | c.113A>C | p.Q38P | Remove H-bond with Glu202 | Misfolding |
| *15* | c.130G>A | p.V44M | Steric clash with Arg347 and Pro351 | Misfolding |
| *16* | c.130G>T | p.V44L | Steric clash with Ala46 and Thr350 | Misfolding |
| *17* | c.131T>C | p.V44A | Cavity | Misfolding |
| *18* | c.134C>T | p.S45L | Remove H-bond with Gln346 and inter-chain H-bond with Ala101 | Dimerisation |
| *19* | c.152G>A | p.R51Q | Disordered loop, remove substrate Interactions? | N/O |
| *20* | c.152G>T | p.R51L | Disordered loop, remove substrate Interactions? | N/O |
| *21* | c.163G>T | p.G55C | Disordered loop, unknown | N/O |
| *22* | c.172G>A | p.E58K | Disordered loop, unknown | N/O |
| *23* | c.184C>A | p.L62M | Disordered loop, unknown | N/O |
| *24* | c.197C>A | p.P66H | Increase flexibility | Misfolding |
| *25* | c.197C>T | p.P66L | Increase flexibility | Misfolding |
| *26* | c.199C>T | p.R67C | Remove H-bond with Asp136 | Misfolding |

| | | | | |
|---|---|---|---|---|
| *27* | c.221T>C | p.L74P | Remove interaction with substrate, decrease flexibility, steric clash with Asn72 and Asn182 | Substrate |
| *28* | c.241G>A | p.A81T | Steric clash with substrate | Substrate |
| *29* | c.247G>A | p.G83R | Decrease flexibility | Misfolding |
| *30* | c.248G>T | p.G83V | Decrease flexibility | Misfolding |
| *31* | c.265T>C | p.Y89H | Remove H-bond with Leu74 | Misfolding |
| *32* | c.265T>G | p.Y89D | Remove H-bond with Leu74, cavity | Misfolding |
| *33* | c.285T>G | p.F95L | Remove interaction with substrate | Substrate |
| *34* | c.290A>G | p.N97S | Remove interaction with substrate and H-bond with Gln188 | Substrate |
| *35* | c.292G>A | p.D98N | None apparent | Polymorphism? |
| *36* | c.292G>C | p.D98H | Remove interaction with substrate and H-bond with Arg80 | Substrate |
| *37* | c.302C>A | p.A101D | Steric clash with Gln346 of interacting chain | Dimerisation |
| *38* | c.308A>G | p.Q103R | Steric clash with His47 of interacting chain | Dimerisation |
| *39* | c.336T>C | p.S112R | Remove H-bond with His114 and Phe117, steric clash with Gln118 | Misfolding |
| *40* | c.337G>A | p.D113N | None apparent | Polymorphism? |
| *41* | c.341A>T | p.H114L | Remove H-bond with Leu116 and inter-chain H-bond with Glu220 | Dimerisation |
| *42* | c.346C>A | p.L116I | Cavity | Dimerisation |
| *43* | c.350T>C | p.F117S | Cavity | Dimerisation |
| *44* | c.354A>C | p.Q118H | Remove H-bond with Pro115 | Dimerisation |
| *45* | c.367C>G | p.R123G | Increased flexibility, remove H-bond with Ala106, Ser108 and Ser121 | Misfolding |
| *46* | c.368G>A | p.R123Q | Remove H-bond with Ala106, Ser108 and Ser121 | Misfolding |
| *47* | c.374T>C | p.V125A | Cavity | Misfolding |
| *48* | c.379A>G | p.K127E | Remove H-bond with Asp96 | Misfolding |
| *49* | c.386T>C | p.M129T | Cavity | Misfolding |
| *50* | c.389G>A | p.C130Y | Steric clash with Leu74 | Misfolding |
| *51* | c.392T>G | p.F131C | Cavity | Misfolding |
| *52* | c.394C>T | p.H132Y | Remove H-bond with Trp134 and Glu146 | Misfolding |

| | | | | |
|---|---|---|---|---|
| *53* | c.396C>A | p.H132Q | Remove H-bond with Trp134 and Glu146 | Misfolding |
| *54* | c.404C>G | p.S135W | Remove H-bond with Cys75 and Arg67, steric clash with Pro183 and His184 | Misfolding |
| *55* | c.404C>T | p.S135L | Remove H-bond with Cys75 and Arg67, steric clash with His184 | Misfolding |
| *56* | c.413C>T | p.T138M | Steric clash with Ser293 | Misfolding |
| *57* | c.416T>C | p.L139P | Decrease flexibility, cavity, disrupt α-helix | Misfolding |
| *58* | c.424A>G | p.M142V | Cavity | Misfolding |
| *59* | c.425T>A | p.M142K | Steric clash with His132 and Val137 | Misfolding |
| *60* | c.425T>C | p.M142T | Cavity | Misfolding |
| *61* | c.428C>T | p.S143L | Remove H-bond with Glu146 | Misfolding |
| *62* | c.442C>G | p.R148G | Increase flexibility, remove H-bond with Asp152 and Asp273 | Misfolding |
| *63* | c.442C>T | p.R148W | Remove H-bond with Asp152 and Asp273 | Misfolding |
| *64* | c.443G>A | p.R148Q | Remove H-bond with Asp152 and Asp273 | Misfolding |
| *65* | c.448G>C | p.V150L | Steric clash with Phe131 | Misfolding |
| *66* | c.452T>C | p.V151A | Loss of hydrophobic interactions | Misfolding |
| *67* | c.460T>C | p.W154R | Cavity | Misfolding |
| *68* | c.460T>G | p.W154G | Increase flexibility, cavity | Misfolding |
| *69* | c.482T>C | p.L161P | Decrease flexibility, cavity | Misfolding |
| *70* | c.493T>C | p.Y165H | None apparent | Polymorphism? |
| *71* | c.496C>G | p.P166A | Increase flexibility | Misfolding |
| *72* | c.499T>C | p.W167R | Steric clash with His301 | Misfolding |
| *73* | c.502G>T | p.V168L | Steric clash with Leu161 and Gly162, | Misfolding |
| *74* | c.505C>A | p.Q169K | Steric clash with Tyr339 and Leu342 of interacting chain, remove H-bond with Trp167, Ile170, and Trp300 | Dimerisation |
| *75* | c.509T>A | p.I170N | Loss of hydrophobic interactions | Misfolding |
| *76* | c.509T>C | p.I170T | Loss of hydrophobic interactions | Misfolding |
| *77* | c.512T>C | p.F171S | Cavity, remove hydrophobic interactions | Dimerisation |
| *78* | c.524G>A | p.G175D | Decrease flexibility, steric clash with Met177 and Pro295 | Misfolding |

| | | | | |
|---|---|---|---|---|
| *79* | c.539G>T | p.C180F | Steric clash with Asn182 and His186 | Substrate |
| *80* | c.541T>G | p.S181A | May alter substrate binding | Substrate |
| *81* | c.542C>T | p.S181F | May alter substrate binding | Substrate |
| *82* | c.547C>A | p.P183T | Increase flexibility | Misfolding |
| *83* | c.550C>G | p.H184D | Remove H-bond with Pro133 | Misfolding |
| *84* | c.552C>A | p.H184Q | Remove H-bond with Pro133 | Misfolding |
| *85* | c.553C>T | p.P185S | Increase flexibility | Misfolding |
| *86* | c.554C>A | p.P185H | Increase flexibility, steric clash with Phe131 and Leu139 | Misfolding |
| *87* | c.554C>T | p.P185L | Increase flexibility, steric clash with Phe131 and Met142 | Misfolding |
| *88* | c.556C>T | p.H186Y | Catalytic residue, removes ability to form covalent intermediate | Substrate |
| *89* | c.563A>G | p.Q188R | Removes interactions with substrate, removes H-bond with Asn97 and Trp191, charge replusion with Arg48 of interacting chain | Substrate/Misfolding |
| *90* | c.563A>C | p.Q188P | Removes interactions with substrate, removes H-bond with Asn97 and Trp191, decrease flexibility, disrupt β-strand | Substrate |
| *91* | c.574A>G | p.S192G | Remove H-bond with Phe194, increase flexibility | Misfolding |
| *92* | c.575G>A | p.S192N | Remove H-bond with Phe194, steric clash with Leu102 and Phe194 | Misfolding |
| *93* | c.580T>C | p.F194L | Cavity, remove hydrophobic interactions | Dimerisation |
| *94* | c.584T>C | p.L195P | Decrease flexibility | Misfolding |
| *95* | c.594T>G | p.I198M | Steric clash with Asp197 | Dimerisation |
| *96* | c.595G>A | p.A199T | Steric clash with Trp167 | Misfolding |
| *97* | c.601C>T | p.R201C | Remove H-bond with Gln38 and Asp39 | Dimerisation |
| *98* | c.602G>A | p.R201H | Remove H-bond with Gln38 and Asp39 | Dimerisation |
| *99* | c.604G>A | p.E202K | Remove interactions with zinc ion, steric clash with Leu37 and Gln38 | Metal |
| *100* | c.607G>A | p.E203K | Remove H-bond with His315 and Trp316 | Misfolding |
| *101* | c.611G>C | p.R204P | Decrease flexibility, disrupt α- | Misfolding |

| | | | | |
|---|---|---|---|---|
| | | | helix | |
| *102* | c.626A>C | p.Y209S | Cavity, remove hydrophobic interactions | Dimerisation |
| *103* | c.626A>G | p.Y209C | Cavity, remove hydrophobic interactions | Dimerisation |
| *104* | c.635A>C | p.Q212P | Decrease flexibility | Misfolding |
| *105* | c.650T>C | p.L217P | Decrease flexibility, cavity, disrupt α-helix | Misfolding |
| *106* | c.652C>G | p.L218V | Cavity, remove hydrophobic interactions | Misfolding |
| *107* | c.658G>A | p.E220K | Remove H-bond with Arg223 and Gln224 and inter-chain H-bond with His114 | Misfolding |
| *108* | c.667C>A | p.R223S | Remove H-bond with Glu220 | Misfolding |
| *109* | c.676C>G | p.L226V | Remove hydrophobic interactions | Misfolding |
| *110* | c.677T>C | p.L226P | Decrease flexibility, remove hydrophobic interactions, disrupt α-helix | Misfolding |
| *111* | c.680T>C | p.L227P | Decrease flexibility, disrupt α-helix | Misfolding |
| *112* | c.687G>T | p.K229N | None apparent | Polymorphism? |
| *113* | c.691C>T | p.R231C | Remove H-bond with Glu225, Glu230 and Glu352 | Misfolding |
| *114* | c.692G>A | p.R231H | Remove H-bond with Glu225, Glu230 and Glu352 | Misfolding |
| *115* | c.697G>C | p.V233L | Steric clash with Lys285 and Leu358 | Misfolding |
| *116* | c.730C>T | p.P244S | Increase flexibility | Misfolding |
| *117* | c.745T>C | p.W249R | Remove hydrophobic interactions | Dimerisation |
| *118* | c.748C>A | p.P250T | Increase flexibility | Misfolding |
| *119* | c.752A>C | p.Y251S | Remove H-bond with Arg357, cavity | Misfolding |
| *120* | c.752A>G | p.Y251C | Remove H-bond with Arg357, cavity | Misfolding |
| *121* | c.756G>T | p.Q252H | Remove H-bond with Thr248 and Trp249, steric clash with Tyr322 | Misfolding |
| *122* | c.769C>A | p.P257T | Increase flexibility, | Misfolding |
| *123* | c.770C>T | p.P257L | Increase flexibility, steric clash with Val261, Arg259 and Gln317 | Misfolding |
| *124* | c.772C>T | p.R258C | Remove H-bond with Ser236 | Misfolding |

| | | | | |
|---|---|---|---|---|
| *125* | c.775C>T | p.R259W | Remove H-bond with Glu266 and Glu271 | Misfolding |
| *126* | c.776G>A | p.R259Q | Remove H-bond with Glu266 and Glu271 | Misfolding |
| *127* | c.785G>C | p.R262P | Decrease flexibility, remove H-bond with Glu266 | Misfolding |
| *128* | c.793C>G | p.P265A | Increase flexibility, remove hydrophobic interactions | Misfolding |
| *129* | c.799C>G | p.L267V | Remove hydrophobic interactions | Misfolding |
| *130* | c.800T>G | p.L267R | Remove hydrophobic interactions, steric clash with Trp239 and Glu271, Leu318 | Misfolding |
| *131* | c.803C>A | p.T268N | Remove H-bond with Glu271 | Misfolding |
| *132* | c.812A>G | p.E271G | Increase flexibility, remove H-bond with Arg259 and Thr268 | Misfolding |
| *133* | c.812G>C | p.E271D | Remove H-bond with Arg259 and Thr268 | Misfolding |
| *134* | c.814C>G | p.R272G | Increase flexibility, remove H-bond with Asp152, Pro265, and Leu267 | Misfolding |
| *135* | c.815G>A | p.R272H | Remove H-bond with Asp152, Pro265, and Leu267, steric clash with Val151, Leu267 and Thr268 | Misfolding |
| *136* | c.833T>A | p.I278N | Remove hydrophobic interactions | Misfolding |
| *137* | c.836T>G | p.M279R | Remove hydrophobic interactions, steric clash with Val151, Trp154, Leu275 and Trp300 | Misfolding |
| *138* | c.844C>G | p.L282V | Remove hydrophobic interactions | Misfolding |
| *139* | c.854A>G | p.K285R | Steric clash with Glu363 | Misfolding |
| *140* | c.855G>T | p.K285N | Remove H-bond with Leu358, Arg359 and Leu361 | Misfolding |
| *141* | c.858T>C | p.Y286H | Remove H-bond with Tyr251 and Thr253 | Misfolding |
| *142* | c.865C>T | p.L289F | Steric clash with Tyr251 | Misfolding |
| *143* | c.866T>G | p.L289R | Steric clash with Tyr251 and Phe290 | Misfolding |
| *144* | c.871G>A | p.E291K | None apparent | Polymorphism? |
| *145* | c.872A>T | p.E291V | None apparent | Polymorphism? |
| *146* | c.881T>A | p.F294Y | Steric clash with Pro324 | Misfolding |

| | | | | |
|---|---|---|---|---|
| *147* | c.883C>A | p.P295T | Decrease flexibility, steric clash with Lys174 and Pro325 | Misfolding |
| *148* | c.922G>A | p.E308K | None apparent | Polymorphism? |
| *149* | c.940A>G | p.N314D | None apparent | Polymorphism? |
| *150* | c.950A>G | p.Q317R | Remove H-bond with Pro257, Arg259, and Val261, steric clash with Met219 and Pro257 | Misfolding |
| *151* | c.951G>T | p.Q317H | Remove H-bond with Pro257, Arg259, and Val261 | Misfolding |
| *152* | c.957C>A | p.H319Q | Remove interactions with zinc ion | Metal |
| *153* | c.958G>A | p.A320T | Steric clash with Leu225 and Met298 | Misfolding |
| *154* | c.959C>T | p.A320V | Steric clash with Leu225 and Met298 | Misfolding |
| *155* | c.961C>T | p.H321Y | Remove interactions with zinc ion, steric clash with Leu37 | Metal |
| *156* | c.967T>C | p.Y323H | Remove H-bond with His321 | Dimerisation |
| *157* | c.967T>G | p.Y323D | Remove H-bond with His321 | Dimerisation |
| *158* | c.968A>G | p.Y323C | Remove H-bond with His301, cavity | Dimerisation |
| *159* | c.970C>T | p.P324S | Increase flexibility | Misfolding |
| *160* | c.974C>T | p.P325L | Increase flexibility | Misfolding |
| *161* | c.980T>C | p.L327P | Decrease flexibility, cavity | Misfolding |
| *162* | c.983G>A | p.R328H | None apparent | Polymorphism? |
| *163* | c.986C>T | p.S329F | Remove H-bond with Thr331 and Val332 | Misfolding |
| *164* | c.989C>T | p.A330V | None apparent | Polymorphism? |
| *165* | c.997C>G | p.R333G | Increase flexibility, remove H-bond with Met117 and Lys334 | Misfolding |
| *166* | c.997C>T | p.R333W | Steric clash with Phe335, remove H-bond with Met117 and Lys334 | Misfolding |
| *167* | c.998G>A | p.R333Q | Remove H-bond with Met117 | Misfolding |
| *168* | c.998G>T | p.R333L | Remove H-bond with Met117 and Lys334 | Misfolding |
| *169* | c.1001A>G | p.K334R | Steric clash with Gln346 | Substrate |
| *170* | c.1006A>T | p.M336L | Cavity | Misfolding |
| *171* | c.1018G>A | p.E340K | Remove H-bond with Lys334, Val337, and Gln346, charge replusion with Lys334 | Substrate |
| *172* | c.1024C>A | p.L342I | Steric clash with Gln169 and His301 of interacting chain | Dimerisation |

| | | | | |
|---|---|---|---|---|
| *173* | c.1030C>A | p.Q344K | Remove inter-chain H-bond with Asp197, steric clash with Phe194 of interacting chain | Dimerisation |
| *174* | c.1034C>A | p.A345D | Steric clash with Thr248 and Met336 | Misfolding |
| *175* | c.1048A>G | p.T350A | Remove H-bond with Asn27 and Gln353 | Misfolding |
| *176* | c.1087G>A | p.E363K | Charge repulsion with Lys281 | Misfolding |
| *177* | c.1103T>C | p.L368P | Disordered C-terminus, unknown | N/O |
| *178* | c.1132A>G | p.I378V | Disordered C-terminus, unknown | N/O |

Missense mutations were obtained from the GALT mutational database (http://arup.utah.edu/database/GALT/GALT_display.php) and their resulting variants were categorised based on their perceived effect of the hGALT structure. Please see Supplementary Figure 8 for further information. N/O: not observed in our hGALT structure.
